# 

**Published:** 1900-10

**Authors:** 


### CONTENTS.

ORIGINAL COMMUNICATIONS. PAGE

Seventeen Supernumerary Teeth. By DWIGHT M. CLAPP, D.M.D., Boston, Mass. 645
Masticating Stress. By B. F. ARRINGTON, D.D.S., Goldsboro, N. C. . . . . . 647
Oxide of Zinc and Eugenol as a Covering to Pulps. By S. B. LUCKIE, D.D.S., Chester, Pa. . . . . . 652
The Successful Dentist. By E. H. RAYMOND, D.D.S. . . . . . 654
Some of the Nerve Remedies. By DR. EDWARD C. BRIGGS, Boston, Mass. . . . 659
A Pathological Suggestion. By JOHN A. McCLAIN, D.D.S., Philadelphia . . . 661

ABSTRACTS AND TRANSLATIONS.

Syphilitic Locolosis Alveolaris (Pyorrhœa Alveolaris). By G. LENOX CURTIS, M.D., New York City . . . . . 664

REPORTS OF SOCIETY MEETINGS.

The New York Institute of Stomatology . . . . . 669
Report of the Foreign Relations Committee of the National Association of Dental Faculties . . . . . 678
Alumni of Harvard Dental School . . . . . 696

EDITORIAL.

The Season for Renewed Effort . . . . . 698

To the Editor of the *Dental Register* . . . . . 701

Dentistry follows the Flag . . . . . 702

Report of Foreign Relations Committee . . . . . 704

NOTES AND COMMENTS . . . . . 705

